# Supplementary material for: Smooth muscle cell estrogen receptor alpha promotes arterial stiffness in the absence of estradiol
Source: bioRxiv. 2026 Mar 5:2026.03.03.709417. Preprint. [Version 1] doi: 10.64898/2026.03.03.709417 (PMC12991074; doi:10.64898/2026.03.03.709417)
Supplement: Supplement 1 [file media-1.pdf]

SUPPLEMENTAL MATERIAL

Smooth muscle cell estrogen receptor alpha promotes arterial stiffness in the absence of estradiol

Casey G. Turner[1,2], Jacqueline Matz[1], Jade Breton[1], Karla C. de Oliveira[1], Rachel Kenney[1], Jennifer Vorn[1], Michelle Zhao[1,3], Jaime Ibarrola[1,4], Qing Lu[1], Gregory Martin[1], Zhe Sun[5], Iris Z. Jaffe[1], Michael A. Hill[5], Jennifer J. DuPont[1*]

Affiliations
[1]Molecular Cardiology Research Institute, Tufts Medical Center, Boston, MA, USA
[2]School of Kinesiology, Auburn University, Auburn, AL, USA
[3]College of Allopathic Medicine, Nova Southeastern University, Fort Lauderdale, FL, USA
[4]Translational Cardiology Unit, Navarrabiomed-Hospital Universitario de Navarra (HUN)-Universidad Pública de Navarra (UPNA), IDISNA, Pamplona, Navarre, Spain
[5]Dalton Cardiovascular Research Center, University of Missouri, Columbia, MO, USA

Short Title: Role of SMC-ERα in arterial stiffening

***Corresponding Author:**

Jennifer J. DuPont

Tufts Medical Center

800 Washington Street, Box 80

Boston, MA 02111

617 636-0620

Jennifer.dupont@tuftsmedicine.org

Keywords: arterial stiffness, aging, estradiol, estrogen receptor alpha, sex differences

SUPPLEMENTAL FIGURES

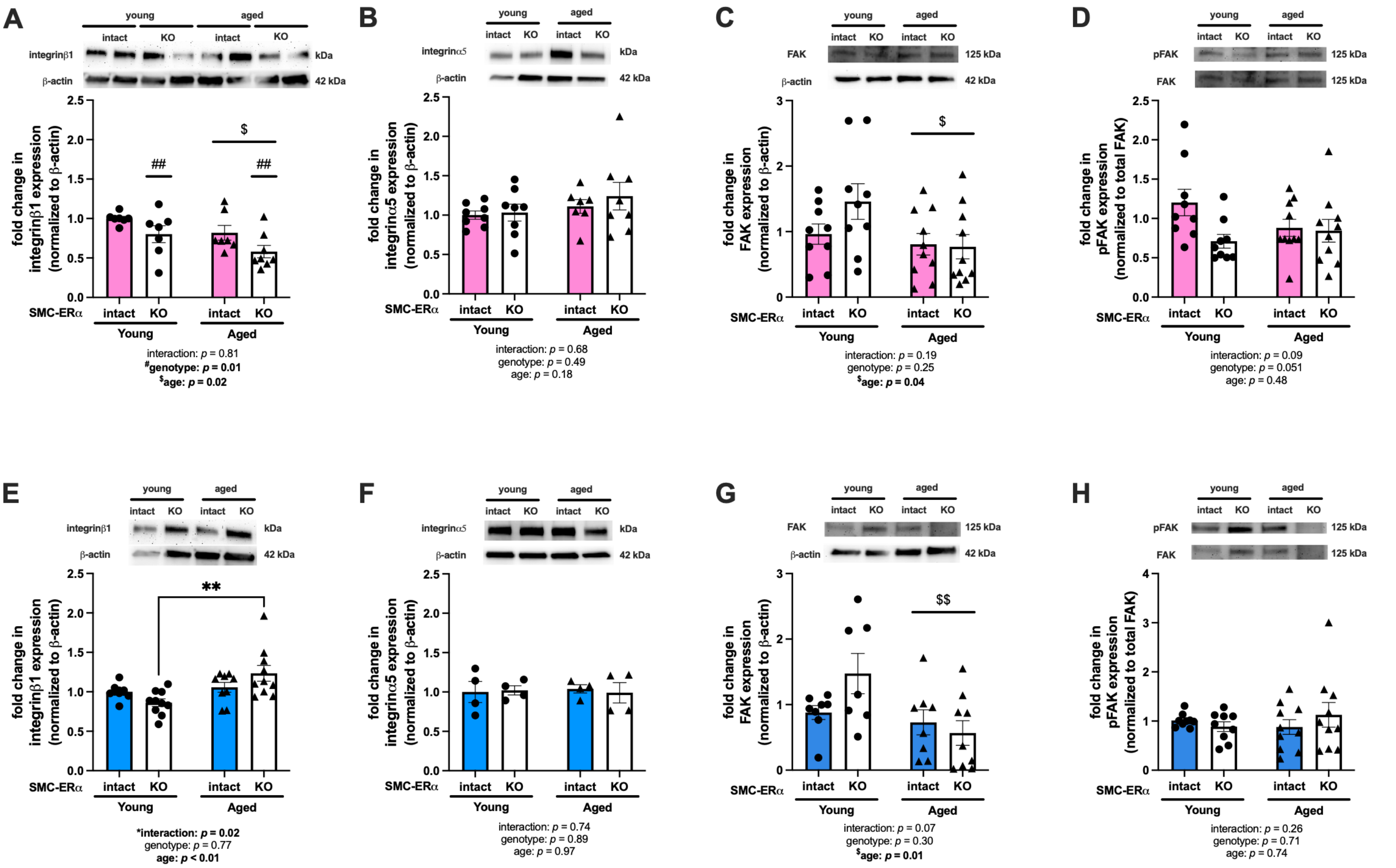

Figure S1. Sex-specific expression of SMC-stiffness associated proteins in aortas from SMC-ERα intact and knockout mice. Whole aortas were collected from young and aged smooth muscle cell (SMC)-specific estrogen receptor α intact and knockout (KO) female and male mice and immunoblotted for expression of proteins associated with SMC stiffness. Expression of integrinβ1 (panel A), integrinα5 (panel B), focal adhesion kinase (FAK, panel C), and FAK phosphorylated at tyrosine 397 (pFAK, panel D) were measured in aortas from female mice and aortas from male mice (panels E-H, respectively). Group differences were assessed by two-way ANOVA (panels A-H). Bonferroni post-hoc testing was used. Data are means ± SEM. ***p*<0.01. Main effect of genotype: ##*p*<0.01. Main effect of age: $*p*<0.05, $$*p*<0.01.

SUPPLEMENTAL TABLES

Table S1. Antibodies used for immunoblotting.

Target antigen	Vendor or Source	Catalog #
Anti-mouse	Cell Signaling	7076
Anti-rabbit	Cell Signaling	7074
β-actin	Proteintech	20536-1-AP
FAK	Cell Signaling	3285
Integrinα5	Abcam	ab226816
Integrinβ1	Abcam	ab179471
MMP2	Santa Cruz	sc-13594
MMP9	Santa Cruz	sc-21733
MMP13	Abcam	ab39012
pFAK	Cell Signaling	3283

Table S2. Animal characteristics — Wildtype ovariectomy experiment.

Variable	SHAM + P	OVEX + P	OVEX + E2
Total n	16	12	16
n with tibia length and kidney mass	9	6	10
n with blood pressure measurements	5	3	5
Δ 24-hr mean arterial pressure (mmHg) (6wk post-surgery minus 1wk post-surgery)	3.0 ± 3.4	2.2 ± 1.7	0.1 ± 1.5
Body mass 6wk post-surgery (g)	22.0 ± 0.4$^{\#}$	26.3 ± 0.5$^{*,\#}$	24.0 ± 0.3
Δ Body mass (g)	2.7 ± 0.3$^{\#}$	6.8 ± 0.4$^{*,\#}$	5.1 ± 0.4
Heart mass (mg)	119 ± 3	125 ± 3	115 ± 1
Left ventricular mass (mg)	82 ± 2	85 ± 2	80 ± 1
Tibia length (mm)	17.3 ± 0.1	17.9 ± 0.2	17.6 ± 0.1
Heart mass (mg) / tibia length (mm)	7.1 ± 0.3	6.8 ± 0.2	6.6 ± 0.1
Left ventricle mass (mg) / tibia length (mm)	4.9 ± 0.2	4.7 ± 0.2	4.5 ± 0.1
Average kidney mass (mg)	140 ± 4	134 ± 6	148 ± 4
Average kidney mass (mg) / tibia length (mm)	8.1 ± 0.3	7.5 ± 0.4	8.4 ± 0.2

E2, estradiol pellet. g, gram. mg, milligram. mm, millimeter. P, placebo pellet. OVEX, ovariectomy surgery. SHAM, sham surgery. wk, week. $^{*}p<0.05$ versus SHAM+P. $^{\#}p<0.05$, versus OVEX+E2.

Table S3. Animal characteristics — SMC-ERα ovariectomy experiment.

Variable	SHAM SMC-ERα-intact	OVEX SMC-ERα-intact	SHAM SMC-ERα-KO	OVEX SMC-ERα-KO
Total n	7	8	6	7
Body mass 6wk post-surgery (g)	22.2 ± 0.5	23.6 ± 0.7*	21.6 ± 0.6	25.4 ± 1.2*
Δ Body mass (g)	2.3 ± .5	4.1 ± .4*	2.6 ± 0.6#	5.3 ± 0.9*,#
Tibia length (mm)	17.9 ± 0.1	18.0 ± 0.1	17.6 ± 0.2	17.9 ± 0.1
Heart mass (mg)	122 ± 5	116 ± 5	119 ± 7	125 ± 5
Heart mass (mg) / tibia length (mm)	6.8 ± 0.3	6.5 ± 0.3	6.8 ± 0.3	7.0 ± 0.2
Left ventricular mass (mg)	83 ± 3	80 ± 3	81 ± 5	85 ± 3
Left ventricle mass (mg) / tibia length (mm)	4.7 ± 0.1	4.4 ± 0.2	4.6 ± 0.3	4.8 ± 0.2
Average kidney mass (mg)	137 ± 4	125 ± 4	125 ± 6	131 ± 6
Average kidney mass (mg) / tibia length (mm)	7.7 ± 0.2	7.0 ± 0.3	7.1 ± 0.3	7.3 ± 0.3

ERα, estrogen receptor alpha. g, gram. KO, knockout. mg, milligram. mm, millimeter. OVEX, ovariectomy surgery. SHAM, sham surgery. SMC, smooth muscle cell. *$p<0.05$, main effect of surgery. #$p<0.05$, main effect of genotype.

Table S4. Animal characteristics — Aging SMC-ERα females.

Variable	YOUNG SMC-ERα-intact	YOUNG SMC-ERα-KO	AGED SMC-ERα-intact	AGED SMC-ERα-KO
Total n	9	9	17	11
Body mass (g)	22.7 ± 0.5	21.2 ± 0.6#	32.1 ± 1.2*	28.5 ± 1.6*,#
Tibia length (mm)	17.6 ± 0.1	17.4 ± 0.1	17.9 ± 0.1*	17.7 ± 0.1*
Heart mass (mg)	117 ± 7	111 ± 6	137 ± 6*	138 ± 5*
Heart mass (mg) / tibia length (mm)	6.7 ± 0.4	6.4 ± 0.3	7.6 ± 0.3*	7.8 ± 0.3*
Left ventricular mass (mg)	80 ± 5	78 ± 4	92 ± 3*	94 ± 4*
Left ventricle mass (mg) / tibia length (mm)	4.5 ± 0.2	4.5 ± 0.2	5.1 ± 0.2*	5.3 ± 0.2*
Average kidney mass (mg)	119 ± 6	118 ± 4	163 ± 7*	163 ± 7*
Average kidney mass (mg) / tibia length (mm)	6.8 ± 0.3	6.7 ± 0.2	9.1 ± 0.4*	9.2 ± 0.4*
Uterus mass (mg)	40 ± 7	34 ± 4	113 ± 14*	73 ± 17*
Uterus mass (mg) / tibia length (mm)	2.3 ± 0.4	1.9 ± 0.2	6.3 ± 0.8*	4.1 ± 0.1*

ERα, estrogen receptor alpha. g, gram. KO, knockout. mg, milligram. mm, millimeter. SMC, smooth muscle cell. *p<0.05, main effect of age. #p<0.05, main effect of genotype.

Table S5. Animal characteristics — Aging SMC-ERα males.

Variable	YOUNG SMC-ERα-intact	YOUNG SMC-ERα-KO	AGED SMC-ERα-intact	AGED SMC-ERα-KO
Total n	10	9	10	8
Body mass (g)	31.4 ± 1.2	28.3 ± 0.7	41.4 ± 2.6*	39.2 ± 1.8*
Tibia length (mm)	18.1 ± 0.1	18.1 ± 0.1	18.3 ± 0.1*	18.4 ± 0.2*
Heart mass (mg)	158 ± 7	146 ± 6	178 ± 11*	160 ± 4*
Heart mass (mg) / tibia length (mm)	8.7 ± 0.4	8.0 ± 0.4	9.7 ± 0.6	8.7 ± 0.3
Left ventricular mass (mg)	104 ± 4	96 ± 4	122 ± 8*	108 ± 5*
Left ventricle mass (mg) / tibia length (mm)	5.8 ± 0.3	5.3 ± 0.2	6.7 ± 0.4*	5.9 ± 0.2*
Average kidney mass (mg)	161 ± 7	150 ± 5$^{\#}$	210 ± 8*	182 ± 5$^{*,\#}$
Average kidney mass (mg) / tibia length (mm)	8.9 ± 0.4	8.3 ± 0.3$^{\#}$	11.5 ± 0.4*	9.9 ± 0.3$^{*,\#}$

ERα, estrogen receptor alpha. g, gram. KO, knockout. mg, milligram. mm, millimeter. SMC, smooth muscle cell. **p*<0.05, main effect of age. $^{\#}p<0.05$, main effect of genotype.
